# *Hepatitis C virus* Broadly Neutralizing Monoclonal Antibodies Isolated 25 Years after Spontaneous Clearance

**DOI:** 10.1371/journal.pone.0165047

**Published:** 2016-10-24

**Authors:** Sabrina J. Merat, Richard Molenkamp, Koen Wagner, Sylvie M. Koekkoek, Dorien van de Berg, Etsuko Yasuda, Martino Böhne, Yvonne B. Claassen, Bart P. Grady, Maria Prins, Arjen Q. Bakker, Menno D. de Jong, Hergen Spits, Janke Schinkel, Tim Beaumont

**Affiliations:** 1 AIMM Therapeutics, Amsterdam, the Netherlands; 2 Department of Medical Microbiology, Section of Clinical Virology, Academic Medical Center, Amsterdam, the Netherlands; 3 Department of Infectious Diseases Research and Prevention, Cluster of Infectious Diseases, Public Health Service of Amsterdam, Amsterdam, the Netherlands; 4 Department of infectious diseases, Academic Medical Center, Amsterdam, the Netherlands; Saint Louis University, UNITED STATES

## Abstract

*Hepatitis C virus* (HCV) is world-wide a major cause of liver related morbidity and mortality. No vaccine is available to prevent HCV infection. To design an effective vaccine, understanding immunity against HCV is necessary. The memory B cell repertoire was characterized from an intravenous drug user who spontaneously cleared HCV infection 25 years ago. CD27+IgG+ memory B cells were immortalized using BCL6 and Bcl-xL. These immortalized B cells were used to study antibody-mediated immunity against the HCV E1E2 glycoproteins. Five E1E2 broadly reactive antibodies were isolated: 3 antibodies showed potent neutralization of genotype 1 to 4 using HCV pseudotyped particles, whereas the other 2 antibodies neutralized genotype 1, 2 and 3 or 1 and 2 only. All antibodies recognized non-linear epitopes on E2. Finally, except for antibody AT12-011, which recognized an epitope consisting of antigenic domain C /AR2 and AR5, all other four antibodies recognized epitope II and domain B. These data show that a subject, who spontaneously cleared HCV infection 25 years ago, still has circulating memory B cells that are able to secrete broadly neutralizing antibodies. Presence of such memory B cells strengthens the argument for undertaking the development of an HCV vaccine.

## Introduction

Hepatitis C is one of the major global public health problems with around 180 million people chronically infected [1] and 500,000 deaths every year from hepatitis C-related complications [2]. Recently, novel antiviral therapies have been shown to be very effective in clearing chronic infections [3–6]. However, *Hepatitis C virus* (HCV) infection often goes unnoticed due to the asymptomatic character of the infection and therefore further spread continues. In addition, these new treatment options are prohibitively expensive and not accessible for most people worldwide. It is therefore unlikely that these treatment options will ultimately minimize the burden of hepatitis C in the world. For this, a vaccine to prevent primary infections is needed. However, such a vaccine is currently not available.

To guide the development of a vaccine, better understanding of the HCV immunity in subjects that spontaneously clear the infection is needed. Spontaneous clearance of the primary HCV infection occurs in 25 to 40% of the infected individuals [7–9]. Importantly, clearance rates after HCV reinfection appear to be increased, as observed among injecting drug users [10]. In addition, the duration and level of viremia is reduced during a reinfection compared to the primary infection [10,11]. These observations suggest that following primary infection, immunological memory against HCV is generated. Strong and broad T cell responses have been associated with clearance of primary HCV infection [12,13] and importantly, HCV specific memory T cells have been detected up to 18 years after infection [14]. In contrast to T cell immunity, less is known about the long-term memory B cell response. A potent and broad neutralizing antibody response against HCV has been associated with spontaneous clearance, especially during the early phase of infection [15,16]. In addition, several broadly neutralizing monoclonal antibodies showed protection against HCV infection in vivo [17,18], suggesting that broadly neutralizing antibody can contribute to viral clearance only when they are present and develop rapidly after infection. However it is unknown if long-term memory B cells capable of producing broadly neutralizing monoclonal antibodies does develop after spontaneous clearance and if these memory B cells can produce these antibodies quickly after reexposure, If so, this will strengthen the argument for undertaking the development of an HCV vaccine eliciting such neutralizing antibody response.

One strategy of vaccine development is based on information on the structure of the virus envelope surface protein in complex with potent broadly neutralizing antibodies. Understanding how these antibodies bind and fix the generally metastable viral envelope fusion proteins have to guide vaccine design. Using such structure based vaccine approach, several groups have been able to elicit neutralizing antibodies against viruses like human immunodeficiency virus, influenza viruses and human respiratory syncytial virus in animal models [19–21]. Such an approach may be applicable for HCV also, especially since the crystal structure of a partly truncated E2 envelope protein was recently solved in complex with an antibody [22,23]. Additional novel antibodies against E1E2 would facilitate development of more and higher quality E1E2 structures.

Most of the human antibodies that broadly bind E1E2 and neutralize HCV, target the E2 protein. Three epitopes have been defined which are located in or around the CD81 binding site: epitope I (amino acids (aa) 412–423), epitope II (aa 434–446) and domain B (aa 523–535) [24–27]. In addition, some broadly reactive and neutralizing antibodies recognize a region adjacent to domain B named domain C or AR2 (aa 538–549) [27,28]. Only few human broadly neutralizing antibodies recognizing E1 have been isolated. Two of these antibodies IgH-505 and IgH-526 target the linear epitope aa 313–327 [29]. More recently, antigenic regions 4 and 5, located at the interface of E1 and E2, were described [30]. Since these antibodies were isolated from chronic HCV patients, it is yet unknown if subjects who spontaneously clear HCV infection make such antibodies and if only these, or additional epitopes are targeted.

We therefore studied the E1E2 specific memory B cell repertoire in an injecting drug user who spontaneously cleared HCV infection. 25 years after viral clearance, we could still find circulating, HCV broadly neutralizing B cells. Four of five isolated antibodies were able to neutralize HCV pseudotyped particles (pp) of different genotypes and targeted epitope II and domain B. Interestingly, one antibody recognized an epitope composed of domain C/AR2 and AR5 suggesting that more than one epitope on E2 may need to be the target for developing an HCV vaccine.

## Materials and Methods

### Ethics Statement

The Amsterdam Cohort Study was conducted in accordance with the ethical principles set out in the declaration of Helsinki and was approved by the Amsterdam Medical Center institutional medical ethics committee. Written informed consent is obtained prior to data collection from every participant [31].

### Subject Selection

Participant D18926 was selected from the Amsterdam Cohort Study on HIV among injecting drug users, a prospective cohort study which started in 1985. Study participants visited the Amsterdam Health Service every 4 to 6 months, where blood was drawn and participants completed a standardized questionnaire about their health, risk behavior, and socio-demographic situation.

Participant D18926 was tested HCV seropositive at cohort entry in 1986 [32] and indicated that he started injecting drugs 1.5 year earlier and continued injecting drugs for 13 years. At cohort entry and during entire follow-up time, HCV RNA was negative as determined at least once for each follow up year by the VERSANT® HCV RNA Qualitative Assay (Siemens Medical Solutions Diagnostics) which has a detection limit of 5 IU/mL. The characteristics of the patient are summarized in Table 1.

**Table 1:** Characteristics of participant D18926.

Demographics	Participant D18926
***Age*** ^***1***^	29 years
***Sex***	Male
***Year of cohort entry***	1986
***Year of last follow up***	2011
***period of injecting drugs***	1985–1999
***HIV status***	Seronegative
***IL28B genotype***	
***SNP rs8099917***	T/G
***SNP rs12979860***	C/T

^1^ at cohort entry, SNP: single nucleotide polymorphism

### Generation of Immortalized B Cells

For this study, 7E7 fresh peripheral blood mononuclear cells were obtained from participant 18926 and memory B cells were immortalized by introducing BCL6 and BCL-xL by retrovirus mediated gene transfer as described previously [33]. In brief, human CD27+IgG+ memory B cells were isolated and after stimulation with CD40 Ligand and interleukin-21, the cells were transduced with a retroviral vector containing the transgenes BCL6, BCl-xL and the marker gene GFP. Transduced B cells were maintained in culture with irradiated CD40 Ligand expressing L-cells and recombinant mouse interleukin-21. The transduced B cells are characterized by the surface expression of the immunoglobulin and secrete immunoglobulin into the culture supernatant.

### Isolation of Cross-Reactive Antibodies

To isolate B cells secreting E1 and / or E2 specific antibodies, we used two approaches: i) immortalized polyclonal B cells were seeded at 100 cells per well in a 96 well plates and maintained in culture for 2 weeks. Antibodies in supernatant of B cell cultures were screened for binding to 293T cells transfected with E1E2 (H77 derived) expression plasmids by flow cytometry using BD Perm/Wash buffer (BD Biosciences) as described by the manufacturer. In brief, fixed and permeabilized E1E2 transfected 293T cells were pre-incubated with B cell supernatants prior to addition of the secondary polyclonal goat anti human Immunoglobulin (Ig)G-PE antibody (Southern biotech) at 1:800 dilution. B cells from positive wells were single cell cultured and after 3 weeks screened for E1E2 binding (H77 derived) and tested for binding to genotypes 1 to 4 by flow cytometry or ii) immortalized B cells were incubated with E2-his from isolate AMS.2b.21 (gt 2b), stained with monoclonal mouse anti-His Alexa Fluor 647 antibody (Qiagen, 1:400 diluted) and positive B cells were bulk sorted, cultured and further enriched for HCV E2 specificity by another round of single cell sorting using E2-his from isolate H77 (gt 1a).

### E1E2 Sequences and Plasmids

E1E2 sequence of isolate H77, (Genbank accession no. AAB67037) was a gift from Dr Jean Dubuisson [34]. E1E2 sequences from the isolates UKN4.11.1, UKN5.15.7 and UKN6.5.340 (accession no. AY734986, EF427672 and AY736194 respectively) [35,36] were synthesized by GeneArt (Invitrogen) and cloned into the pcDNA3.1 Zeo(+) vector (Invitrogen).

From plasma of local chronically infected patients, AMS E1E2 sequences were isolated using the Boom extraction method [37]. In brief, RNA was purified from stored plasma, cDNA was synthesized and the sequences of E1 and E2 were obtained (AMS.1b.2, AMS.2b.21, AMS.3a.26 and AMS.4d.8 see Genbank accession no. KR094962, KR094963, KR094964 and KR094965).

To determine which amino acids of E2 are crucial for antibody binding, the H77 E1E2 sequences were synthesized with single alanine mutations. 25 E2 mutants were generated either by GeneArt (Invitrogen), by using primers coding for the specific mutation (Biolegio) or by use of the QuikChange II XL Site-Directed Mutagenesis Kit (Agilent). If the residue of interest was alanine, it was substituted by a glycine.

To generate HCVpp viruses 2 plasmids are needed next to a vector expressing the E1E2 sequences: the phCMV vector containing the MLV gag/pol and the phCMV packaging vector encoding the Luciferase gene (both are a gift from Dr Jean Dubuisson).

### Soluble E2 Protein and Antibodies

To obtain purified E2 proteins, the E2 ectodomain (aa 384–717) from isolate H77 and isolate AMS.2b.21 containing a His6-tag (E2-his) were cloned into the pCPEO-ST vector and produced in 293T cells (directly acquired from ATCC, CRL-11268). After harvest of the culture supernatant, the E2-his was purified using a HisTrap FF column on an ÄKTA Explorer 10s (GE healthcare).

From broadly reactive B cells the VH and VL were sequenced, cloned and recombinant antibody produced [38]. In brief, total RNA was isolated from B cells and after generation of cDNA, the sequences were obtained from the heavy and light chain by PCR. To produce recombinant antibodies, the VH and VL sequences were cloned onto the human IgG1 and kappa constant regions in pcDNA3.1 (Invitrogen). 293T/17 cells were transiently transfected and recombinant antibodies were purified using the MabSelect SuRe column (GE Healthcare) on an ÄKTA explorer. The H53 antibody [39] was a gift from Dr Jean Dubuisson.

### Enzyme-Linked Immunosorbent Assay (ELISA)

Binding of recombinant antibodies to E1 and/or E2 was determined using an ELISA in which (a) the aa 384–717 E2 ectodomain with a His6 tag or (b) a 1% triton lysate of 293T/17 cells transfected with E1E2 was captured on plates coated with *Galanthus nivalis* lectin (GNA) (Sigma) as described previously [30]. In brief, pre-coated plates were blocked with 1% fish skin gelatin (Sigma) and 0.05% tween in phosphate buffered saline before antibodies were added. To detect bound antibodies, the plates were incubated with horseradish peroxidase conjugated polyclonal goat anti-human IgG (Jackson, 1:2500 diluted). Bound antibodies were detected using 3,3’, 5,5’ tetramethyl benzidine (Sigma) and the reaction was stopped using H_2_SO_4_. Optical density (OD) at 450nm was measured with an EnVision Multilabel Reader (PerkinElmer).

### Neutralization Assay with HCVpp

Neutralization assays were performed as described previously [40]. In brief, HCVpp expressing E1E2 from isolates H77 (gt 1a), AMS.1b.2 (gt 1b), AMS.2b.21 (gt 2b), AMS.3a.26 (gt 3a), UKN4.11.1 (gt 4) and AMS.4d.8 (gt 4d), were incubated for 1 hour at 37°C with 50 to 0.0008 μg/mL of antibody before being spin-inoculated (2000 x g for 45 minutes) onto Huh-7 cells (a gift from François Cosset) seeded one day in advance. After 3 days, the Huh-7 cells were lysed using Bright-Glo™ Luciferase Assay System (Promega Corporation). Neutralization was determined by the reduction in luciferase activity compared to background and control samples. Only HCVpp supernatants, which generated luciferase activity 10 times the background, were included (S1 Fig). The background was subtracted from the relative light value of each well. Antibody neutralization activity was calculated using the mean relative light unit from wells incubated without antibodies. Antibody concentration to reach 50% neutralization or (IC_50_) and antibody concentration to reach 90% neutralization (IC_90_) were determined using nonlinear regression analysis. The data were analyzed using Prism software (GraphPad). VSV-G pp was used as control for aspecific effect.

### Competition Assays by Flow Cytometry

To determine antibody inhibition of CD81 binding to E2, an intracellular staining was performed using BD Perm/Wash buffer (BD Biosciences) as described by the manufacturer. In brief, fixed and permeabilized E1E2 transfected 293T cells were pre-incubated with antibody prior to addition of the large extracellular loop of CD81 (CD81-LEL) tagged mouse Fc (Sino biological). After washing, the polyclonal Alexa Fluor 647 conjugated goat anti-mouse Fc antibody (Jackson, 1:12800 diluted) was added and fluorescence was measured using a BD FACSCanto ^TM^ II flow cytometer (BD Biosciences). The data were analyzed using FlowJo^TM^ software (FlowJo). The inhibition of CD81 binding to E1E2 was calculated using the percentage of Alexa Fluor 647 positive cells from wells incubated without antibodies.

To determine antibody competition for E2 binding, flow cytometry assays were performed in a similar fashion as described above with the following adaptation: Fixed and permeabilized E1E2 transfected 293T cells were pre-incubated with non-labeled antibody prior to addition of E1E2 specific antibodies AR3B, AR4A or AT12-011, which were conjugated with Alexa Fluor 647.

### Surface Plasmon Resonance (SPR) Analysis

Antibody affinity for the HCV envelope glycoprotein E2 was determined using SPR as described previously [38]. In brief, an amine-specific EasySpot gold-film gel-type SPR-chip (Ssens BV) was spotted with anti-HCV antibodies. After deactivation of the sensor and washing, concentration series of HCV E2-his was injected to measure binding kinetics. Data were analyzed using Sprint software (IBIS Technologies BV). K_D_ was calculated as dissociation constant / association constant.

To determine antibody-binding competition on E2, SPR assays were performed in a similar fashion as described above. But, after immobilizing one antibody on the chip, followed by E2 capture, the second antibody was injected. If no signal was obtained with the second antibody both antibodies recognized an identical epitope or at least an epitope in close proximity.

## Results

### Isolation of E1E2 Specific Monoclonal Antibodies

In 2011, approximately 25 years after the estimated date of HCV acquisition, peripheral blood mononuclear cells were obtained from fresh blood of participant D18926. CD27+IgG+ memory B cells were isolated (8.4E5 cells) and immortalized. B cells specific for E1E2 were isolated (i) by screening 7.7E05 cells for binding to 293T cells expressing E1E2 from gt 1a (H77) by flow cytometry and (ii) by two rounds of cell sorting with E2 from AMS.2b.21 (gt 2b) followed by sorting with E2 from H77 (gt 1a). During screening, monoclonal B cell cultures were also tested for binding to a panel of E1E2 proteins from gt 1a (H77), 1b (AMS.1b.2), 2b (AMS.2b.21), 3a (AMS.3a.26) and 4a (UKN4.11.1). Antibodies AT12-007, -009, -010, -011 and AT13-021 showed binding to E1E2 from genotypes 1,2, 3 and 4. Since AT12-008 and AT12-012, showed binding to E1E2 from gt 1a only we discontinued characterizing them (S2 Fig).

**Fig 1:**
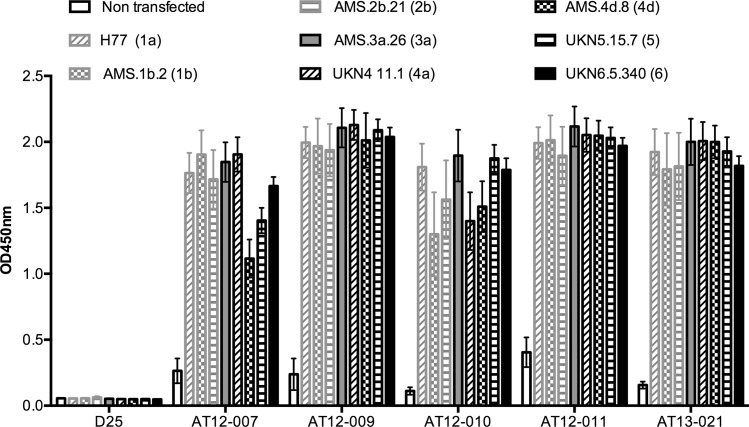
Breadth of cross-reactive antibodies against E1E2 glycoproteins. Antibodies were tested for binding to E1E2 protein derived from different genotypes by ELISA. E1E2 containing cell lysates were incubated on GNA pre-coated wells before antibodies were added at 1 μg/mL. A lysate from non-transfected cells and the RSV F protein specific antibody D25 were used as negative controls. The y-axis indicates the mean optical density (OD) at 450 nm and the errors bars represent one standard deviation (SD). E1E2 sequences are indicated between brackets and assays were performed in duplicate.

The variable regions VH and VL of the five cross-reactive antibodies were cloned onto the human IgG1 and kappa constant regions to produce recombinant antibodies. Sequence characteristics of antibodies are described in Table 2. The recombinant monoclonal antibodies AT12-007, AT12-009, AT12-010, AT12-011 and AT13-021 were first tested for binding to a panel of E1E2 derived from six different genotypes 1a (H77), 1b (AMS.1b.2), 2b(AMS.2b.21), 3a (AMS.3a.26), 4 (UKN4.11.1), 4d (AMS.4d.8), 5 (UKN5.15.7) and 6 (UKN6.5.340) by ELISA at 1 μg/mL (Fig 1). All recombinant antibodies bound to E1E2 from all genotypes tested.

**Table 2:** Antibody sequence characteristics.

	AT12-007	AT12-009	AT12-010	AT12-011	AT13-021
***Isotype***	IgG1	IgG1	IgG1	IgG1	IgG1
***V gene***	VH1-24	VH1-69	VH1-69	VH1-69	VH1-69
***Number of mutations in VH (nt)***	17 (9R/8S)	34 (26R/8S)	23 (16R/7S)	36 (27R/9S)	20 (13R/7S)
***Number of mutations in VL (nt)***	19 (13R/6S)	17 (12R/5S)	20 (16R/4S)	8(7R/1S)	9(8R/1S)
***CDR3H length (aa)***	17	27	20	13	19

Antibody heavy and light chain genes sequences were analyzed using the IMGT/V-QUEST database. VH: variable domain of the heavy chain, VL: variable domain of the light chain, R: replacement mutations, S: silent mutations nt: nucleotide, CDR: complementarity determining region, aa: amino acids.

### Neutralization Activity of Monoclonal Antibodies

**Fig 2:**
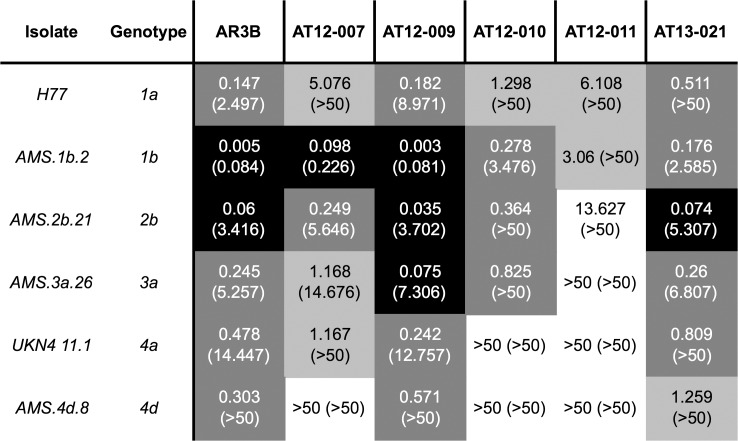
Antibody neutralization of HCVpp. HCV antibody neutralizing activity was determined by pre-incubation of HCVpp with antibodies (50 μg/mL with to 0.0008 μg/mL) before being added to Huh-7 cells. 50 and 90% inhibitory concentrations (IC_50_ and IC_90_ in μg/mL) were determined using non-linear regression analysis. The grey colors indicate the range of neutralization: an IC_50_ <0.1 μg/mL (black), 0.1–1 μg/mL (dark grey), 1–10 μg/mL (light grey) and >50 μg/mL (white). IC_90_ are in parentheses. The mean value of three triplicate experiments is shown.

Antibody neutralization was tested using the HCVpp system expressing gt 1 to 4. Antibodies AT12-009 and AT13-021 neutralized all HCVpp within a concentration range of 0.001 to 0.94 μg/mL, while AT12-007, AT12-010 and AT12-011 had a more restricted neutralization. AT12-007 and AT12-010 neutralized all HCVpp except gt 4d and/or 4a, while AT12-011 neutralized HCVpp from gt 1a, 1b and 2b only (Fig 2 and S3 Fig).

### Interference with CD81 Binding

**Fig 3:**
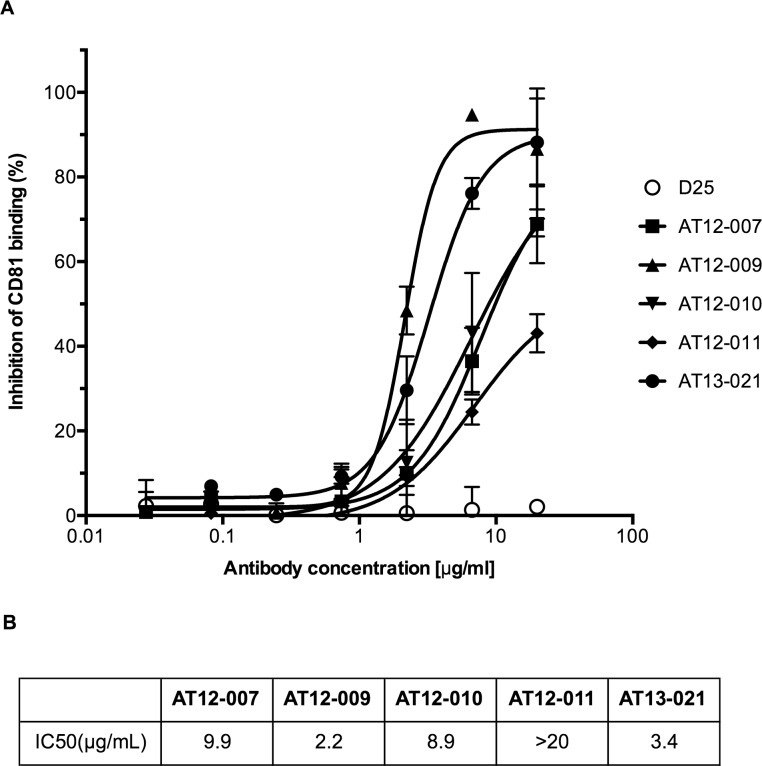
Antibodies compete CD81 binding to E2. Cells transfected E1E2 from isolate H77 were pre-incubated with different concentrations of the indicated antibodies before CD81-LEL at 0.05 μg/mL was added. (A) D25 was used as isotype negative control. The y-axis indicates the percentage inhibition of CD81 and the errors bars represent one SD. (B) Antibody concentration to reach 50% inhibition of CD81 binding (IC_50_) in μg/mL. The IC_50_ was determined using non-linear regression analysis. The assay was performed in duplicate and at least repeated once.

Since CD81 is one of the major HCV receptors and the majority of the neutralizing antibodies interfere with E2 binding to CD81, we performed a CD81 inhibition assay and found that all our antibodies also interfered with CD81 binding to E1E2 although potencies differed between antibodies (Fig 3). As for the neutralization assay, AT12-009 and AT13-021 most efficiently inhibited CD81-LEL binding to E1E2, with IC_50_ values of 2.2 and 3.4 μg/mL respectively.

### Binding Properties

**Fig 4:**
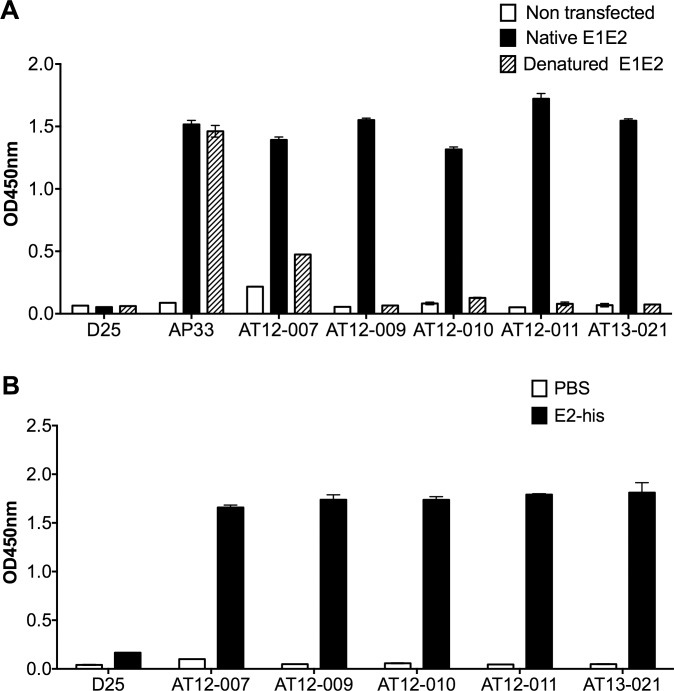
Antibodies recognize non-linear epitopes on soluble E2. (A) Antibody binding to denatured E1E2 was tested by ELISA. A cell lysate of 293T cells transfected with E1E2 (H77) was treated with 20 mM Dithiothreitol and 0.4% Sodium dodecyl sulfate (denatured E1E2) or directly added on GNA pre-coated plate (native E1E2). Subsequently the antibodies were added at 1 μg/mL. A lysate of non-transfected cells, a native E1E2 cell lysate, AP33 an antibody specific for a linear epitope on E2 and D25 were used as negative and positive controls. (B) The binding of antibodies to H77 derived E2 was tested by ELISA. The antibodies (1 μg/mL) were added to wells pre-coated with His6 tagged E2 (E2-his). Phosphate buffered saline (PBS) coated wells and D25 were used as negative controls. In A) and B) the y-axis indicates the mean Optical density (OD) at 450nm and the errors bars represent one SD. The assay was performed in duplicate wells and repeated in at least one separated experiment.

To determine if the epitopes targeted by the antibodies are linear or non-linear, we tested antibody binding at a concentration of 1 μg/mL to native E1E2 glycoproteins captured on a GNA coated ELISA plate vs. binding to denatured E1E2 proteins (isolate H77). Fig 4A shows that all five antibodies bind a conformation dependent, non-linear epitope. The AP33 control antibody, which is directed against a linear E2 epitope, is the only antibody not affected by the denaturation of the E1E2 proteins. In addition, all five antibodies were directed against an epitope present on soluble E2, as they all bound to soluble recombinant E2 from isolate H77 expressed without E1 (Fig 4B). Finally, we determined the equilibrium dissociation (K_D_) of the antibodies for H77 and AMS.2b.21 derived E2 by SPR analysis (Table 3 and S4 and S5 Figs). Similar to the results of the neutralization assay, AT12-007 and AT12-010 showed preferred binding to isolate AMS.2b.21 (3.8 nM and 22.8 nM respectively) compared to binding to E2 from isolate H77 (44 nM and 39.3 nM respectively). The antibodies AT12-009 and AT13-021, with the most potent and broad neutralization profiles, had higher affinities than AT12-007 and AT12-010 (1.3 nM and 14.2 nM for E2 gt 1 and 2.3 nM and 2 nM for E2 gt 2). Interestingly, AT12-011 showed a more than four times higher affinity for E2 (0.2 nM for gt 1 and 0.3 nM for gt 2b) than the other antibodies.

**Table 3:** Affinity of the antibodies for HCV E2.

		AT12-007	AT12-009	AT12-010	AT12-011	AT13-021
*E2 genotype 1a*	K_a_ (10^−5^ s^-1^ M^-1^)	4.6 (± 0.9)	8.6 (± 0.8)	3.6 (± 0.5)	13.6 (± 3.9)	2.9 (± 0.6)
	K_d_ (10^−5^ s^-1^)	184 (± 31)	11 (± 1.6)	142 (± 43)	3.9 (± 1.7)	36 (± 8.9)
	**K**_**D**_ **(nM)**	**44 (± 16)**	**1.3 (± 0.3)**	**39.3(± 6.4)**	**0.3 (± 0.1)**	**14.2 (± 6.2)**
*E2 genotype 2b*	K_a_ (10^−5^ s^-1^ M^-1^)	5.0 (± 0.3)	8.9 (± 1.6)	4.6 (± 1.2)	8.8 (± 3.4)	5.3 (± 0.4)
	K_d_ (10^−5^ s^-1^)	19 (± 0.6)	20 (± 1.5)	98 (± 4.2)	1.1 (± 0.5)	10 (± 0.3)
	**K**_**D**_ **(nM)**	**3.8 (± 0.1)**	**2.3 (± 0.5)**	**22.8(± 4.2)**	**0.2 (± 0.1)**	**2.0 (± 0.1)**

Affinity of the antibodies for HCV E2 was measured for E2 (A) from isolate H77 (genotype 1a) (B) E2 from isolate AMS.2b. 21 (genotype 2b) by direct SPR. Antibodies were immobilized on a chip before injecting a concentration series of E2-his (0.1 to 2.0 μg/ml). The assays were performed in triplicate and the mean of equilibrium dissociation constant (K_D_) is shown (+/- variance). *k*_a_: association constant, *k*_d_: dissociation constant.

### Epitope Mapping

**Fig 5:**
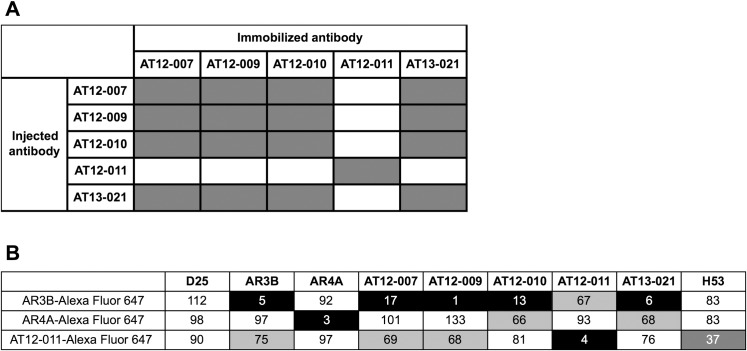
Antibody competition. (A) Antibody competition assay was performed by SPR using H77 derived E2-his. When the antibodies (indicated in rows) were immobilized on the chip and had captured E2, a second antibody (indicated in columns) was injected. Absence of binding after injection of the second antibody indicated the antibodies competed for E2 binding (grey); if the injected antibody bound antibody-captured E2, this was considered to be not competing (white). The assay was performed in triplicate and repeat in one separated experiment. (B) Antibody competition assay was performed by flow cytometry using cells transfected with E1E2 from isolate H77. Cells were pre-incubated with 20 μg/mL of the indicated antibodies before Alexa Fluor 647 conjugated antibodies at their EC_50_ were added. The percentage of binding was calculated using the percentage of Alexa Fluor 647 positive cells from wells incubated without antibodies. The gray scale indicates the relative binding level: white 100% -75%, light grey 75% -50%, dark grey 50% -25% and black 25%–0%. D25 was used as isotype negative control. The mean value of three duplicate experiments is shown.

To identify the antibody binding epitope on E2, SPR competition experiments were performed using His6 tagged E2 (E2-his) from isolate H77. As shown in Fig 5A, AT12-007, -009, -010 and AT13-021 compete for binding to E2. In contrast, AT12-011 did not compete for binding with any other antibody, indicating that AT12-011 binds a different epitope on E2. The SPR curves are shown in S6 Fig.

Competition assays were performed using the well-characterized antibodies AR3B, AR4A and H53. AR3B binds epitope II (aa 434–446) and domain B (aa 523–535), AR4A binds the stem region of E2 and the N terminal region of E1, while H53 binds the AR1 domain which is comprised of the two regions: a first one from aa 483 to aa 485 and second one from aa538 to aa 549 (domain C /AR2) [27,28,30]. Fixed E1E2 transfected 293T cells were pre-incubated with non-labeled antibody prior to addition of AR3B, AR4A or AT12-011 directly conjugated with Alexa Fluor 647. AT12-007, -009, -010 and AT13-021 fully inhibited the binding of AR3B indicating that the epitope of these antibodies comprises the AR3 domain (Fig 5B). However, AT12-010 and AT13-021 binding was also partial inhibited by AR4A, suggesting that their epitopes are not restricted to the AR3B domain only. Binding of AT12-011 was partially inhibited by AR3B but inhibition was more pronounced by H53, suggesting that the epitope of AT12-011 partially overlaps with AR1 and is in close proximity to AR3.

**Fig 6:**
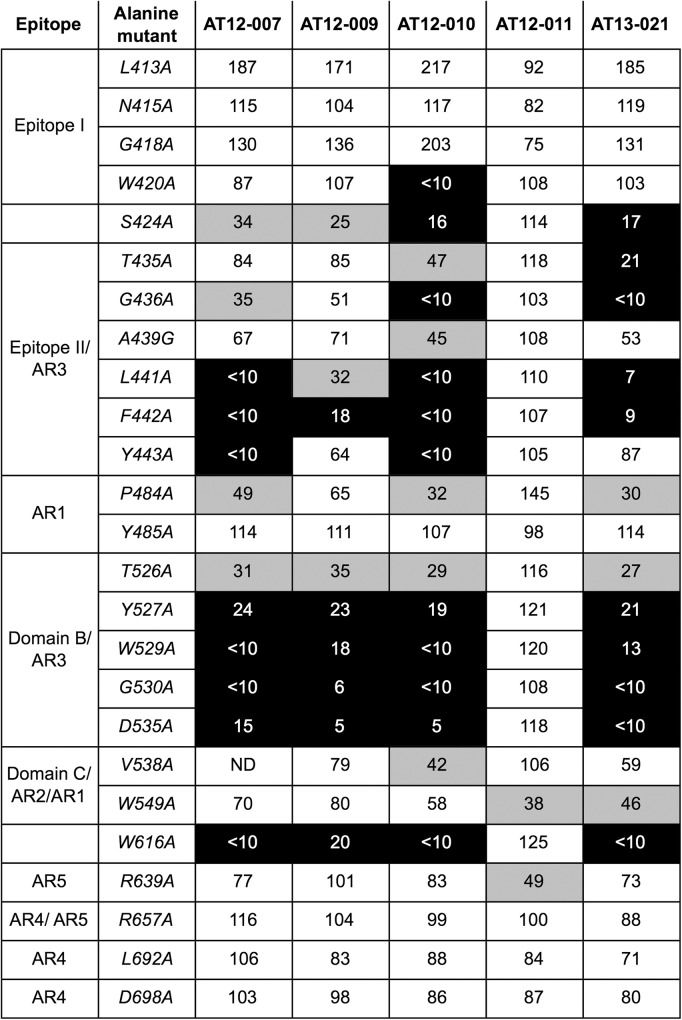
Antibody epitope mapping. To determine the more exact antibody-binding epitope single alanine amino acid substitutions were introduced in the E1E2 sequence from isolate H77 and antibody binding was tested by ELISA. E1E2 containing cell lysates were incubated on GN lectin pre-coated wells before adding an antibody (1 to 0.0002 μg/mL). Antibody concentrations needed to get 50% binding (EC_50_) was determined using non-linear regression analysis. The relative binding was calculated by dividing the EC_50_ obtained on wild-type protein versus alanine-mutant protein times 100. The gray scale indicates the relative binding level: Black 0% -25%, grey 25% -50% and white 50% -150%. The E2 mutants (rows) were designated X123Y where 123 is the residue position, X indicates the wild-type amino acid residue in H77 and Y indicates the replacing amino acid. The EC_50_ which could not be calculated because of very weak binding were indicated by <10%. The data are the mean values of two experiments performed in duplicate.

To determine which amino acid residues in E2 are important for antibody binding, we generated a panel consisting of 25 H77 E2 alanine mutants with mutations at positions shown to affect binding of neutralizing antibodies [28,30,41,42]. Cell lysates of 293T cells transfected with mutant forms of E1E2 were incubated on GNA pre-coated plates before antibodies were added (1000–0.2 ng/mL). The concentrations at which antibodies reach 50% binding or half maximal effective concentration (EC_50_) were determined using non-linear regression analysis. Fig 6 presents the percentage of antibody binding to E2 mutants compared the binding to the wild-type protein, which was calculated by dividing the EC_50_ obtained with wild-type E1E2 sequence by the EC_50_ obtained with the mutant form times 100. In agreement with the competition data, AT12-007, -009, -010 and AT13-021 showed reduced binding (≤25%) to alanine mutants located in epitope II, domain B and W616. Differences in E2 binding between these 4 antibodies were only observed in epitope I and II. For example, AT12-009, the most potent neutralizing antibody seemed less dependent on residues in epitope II. On the contrary, AT12-010, with a more restricted neutralization profile, had a relatively large binding epitope including residues W420 (epitope I), S424, A439, Y443 (epitope II), P484 (AR1) and V538 (domain C/AR2). No reduction in binding was observed using alanine mutants specific for AR4 and epitope I, except for AT12-010 which showed reduced binding to W420. In contrast to AT12-007, -009, -010 and AT13-021, the binding of AT12-011 was not affected by any of the alanine mutants in epitope I, epitope II, domain B or AR4. Consistent with the results of the competition assay, AT12-011 did show reduced binding (≤50%) to alanine mutants at position W549 and R639 located in AR1/ domain C /AR2 and AR5 respectively. These data suggest that AT12-011 binds an epitope which is at least comprised of amino acid also affecting binding of AR5 and AR2/domain C located in close proximity to AR3 binding.

## Discussion

In this study, we analyzed the memory B cells of an injecting drug user who spontaneously cleared a HCV infection 25 years ago and had not been injecting drugs for the last 12 years. Our main finding is that at the time we obtained fresh blood we could retrieve circulating memory B cells that could produce broadly neutralizing antibodies specific for HCV E2. Although we showed that a long-term memory B cells can develop against the HCV E1E2 glycoproteins, it is still unclear if all subjects who spontaneously clear HCV infection develop such B cell memory.

From this subject who spontaneously cleared HCV infection, we could isolate five broadly neutralizing antibodies, which may or may not have been part of the primary immune response against HCV in the past. Although the functions and roles of the antibodies in HCV clearance and infection are still unclear, it was shown that the development of a neutralizing antibody response against E1E2 glycoproteins during the early phase of infection is associated with viral clearance [15,16]. In addition, several broadly neutralizing antibodies showed protection against HCV infection in vivo [17,18] suggesting that broadly neutralizing antibody contribute to viral clearance. Therefore, it is tempting to speculate that the broadly neutralizing antibodies we retrieved from the memory B cell pool 25 years after primary infection may have contributed to viral clearance.

It should be noticed that the subject reported injecting drugs for 13 years following clearance of primary infection and was part of a population of drug users with a HCV antibody prevalence of 84% and an incidence of 6–27 initial infections per 100 person-years [32]. In addition, he indicated having shared needles once in a cohort where borrowing is underreported [43]. Taken together, it is not unlikely that this subject, during his drug use career, was re-exposed to HCV and possibly may have had reinfections, which occurred frequently in this cohort [44]. Because of possible HCV reexposures, it is unknown if the subject developed the memory B cells capable of producing the broadly neutralizing antibodies during primary infection or upon a reexposure.

All antibodies are able to inhibit the binding of E1E2 to the HCV receptor CD81. In addition, two of the broadly reactive antibodies, AT12-009 and AT13-021, are able to neutralize HCVpp of genotypes 1 to 4 with high potency. Due to commercial restrictions, we however did not test these antibodies in the HCV cell culture system (HCVcc). But since Urbanowicz et al. showed that neutralization potency correlated between the HCVpp and the HCVcc system [45] we presume that the AT antibodies will also show neutralization in the HCVcc system. However, the potency of these antibodies may differ between the systems.

Broadly neutralizing antibodies, retrieved from chronically infected patients, [28,30,41,46,47] generally target epitopes located in E2 (e.g. epitope I, epitope II, domain B and the AR4 and AR5 epitopes). Recently the E2 structure and the spatially organization of epitope II and domain B were revealed [22]. Epitope II and domain B overlap and form the epitope for antibodies like AR3. The importance of this epitope was shown by De Jong et al. who demonstrated that combinations of AR4 and AR3 antibodies not only protect mice from HCV infection but can also cure infection [17]. Since antibodies against these antigenic domains have been isolated from chronically infected patients [28], it remains unclear if antibodies against these epitopes are also elicited by subjects who spontaneously cleared HCV infection. In this study 4 out of 5 antibodies target residues in epitope II and antigenic domain B, identical epitopes recognized by antibodies discovered from chronic patients. Thus, it seems that both chronically infected patients and those who clear the infection can elicit antibodies against the same epitopes. As suggested by Pestka et al. [15], this could indicate that timing of the immune response is important and that failure to clear the infection is related to a delay in the development of neutralizing antibodies rather than to the type of epitope targeted.

For in vivo protection, De Jong et al. showed that antibody AR4 which targets an epitope located at the interface of the stem-region of E2 and the N-terminal region of E1, was needed to get full protection in mice, while protection using only AR3 antibodies was incomplete [17,30]. In our study we did not recover antibodies specific for this stem-region. Similarly to the CBH-7 antibody which also targets domain C [26,48], AT12-011 binding to E2 is very efficient but it is not broadly neutralizing (besides gt 1 and 2), suggesting that its epitope is expressed differently on virus particles of the different genotypes or the epitope’s function is different between genotypes. In addition, Swan *et al*. showed that domain C specific antibodies can found in sera and that chronically infected patients with reduced liver fibrosis in general seem to have domain B and C specific antibodies [49]. Collectively, this suggests that several epitopes on E1E2 may need to be targeted by a HCV vaccine.

In conclusion, we demonstrated that 25 years following spontaneous clearance broadly neutralizing antibodies from the memory B cell pool specific for E2 could be detected in a subject who spontaneously cleared HCV infection and remained injecting drugs for another 13 years. The existence and presence of such a long-term memory B cells strengthens the argument for undertaking the development of an HCV vaccine.

## Supporting Information

S1 Fig Infectivity of HCV pseudoparticles (pp). HCVpp carrying HCV envelope proteins from genotypes 1 to 4 were kept for 1 hour at 37°C before being added to Huh-7 cells. To determine the background infectivity of the assay, supernatant of 293T cells transfected only with phCMV-gag/pol and phCMV-Luciferase (no envelope glycoprotein control) was used. The mean value of a triplicate experiment is shown and the errors bars represent one standard deviation (SD).
(TIFF)

S2 Fig Screening for cross-reactive antibodies to E1E2. B cells specific for E1E2 were isolated using two strategies. (A) B cell supernatants were screened for binding to 293T cells expressing E1E2 by flow cytometry. E1E2 transfected 293T cells were permeabilized before adding B cell supernatants containing antibodies. First, B cell supernatant were screened for binding to 293T cells expressing E1E2 from gt 1a (H77) (top panel). Then, positive B cell supernatants were tested for binding E1E2 from genotype 1 to 4 (bottom panel). Non-transfected expressing 293T cells were used as control for non-E1E2 specific binding. (B) B cells specific for E1E2 were isolated with two rounds of cell sorting with E2 (AMS.2b.21, genotype 2b) followed by E2 from H77, genotype 1a. First, B cell supernatant of AT13-021 was tested binding on gt1a H77 E2 ELISA (top panel). Supernatant containing AT13-021 was tested for binding to E1E2 proteins derived from genotype 1 to 4 by ELISA (bottom panel). D25 was used as isotype control.
(TIF)

S3 Fig Antibody neutralization curves. HCV antibody neutralizing activity was determined by pre-incubation of (A) VSV-G pp or HCVpp from isolates (B) H77 (genotype 1a), (C) AMS.1b.2 (genotype 1b), (D) AMS.2b.21 (genotype 2b), (E) AMS.3a.26 (genotype 3a), (F) UKN4.11.1 (genotype 4) and (G) AMS.4d.8 (gt4d) with antibodies (50 μg/mL to 0.0008 μg/mL) before being added to Huh-7 cells. The mean value of two triplicate experiments is shown and the errors bars represent one standard deviation (SD).
(TIFF)

S4 Fig SPR curve fits of the binding of antibodies to E2 genotype 1a. A concentration series of H77 derived E2-his (0.1–2.0 μg/mL) is injected in duplicate over an antibody-coated SPR chip. SPR data is shown as black curve. SPR curves are fit to a 1:1 binding model to obtain kinetic constants; curve fits are shown as grey lines.
(TIF)

S5 Fig SPR curve fits of the binding of antibodies to E2 genotype 2b. A concentration series of E2-his from genotype 2b isolate AMS.2b.21 (0.1–2.0 μg/mL) is injected in duplicate over an antibody-coated SPR chip. SPR data is shown as black curve. SPR curves are fit to a 1:1 binding model to obtain kinetic constants; curve fits are shown as grey lines.
(TIF)

S6 Fig Antibody competition by SPR. E2-his (2.0 μg/mL) is first injected over an antibody-coated SPR chip. After binding E2-his, a second antibody is injected. If the second antibody binds to the antibody-E2 complex, this indicates that the second antibody has a different epitope than the first antibody.
(TIF)
