# Supplementary figures and images for: Hepatitis C virus Broadly Neutralizing Monoclonal Antibodies Isolated 25 Years after Spontaneous Clearance
Source: PLoS One. 2016 Oct 24;11(10):e0165047. doi: 10.1371/journal.pone.0165047 (PMC5077102; doi:10.1371/journal.pone.0165047)

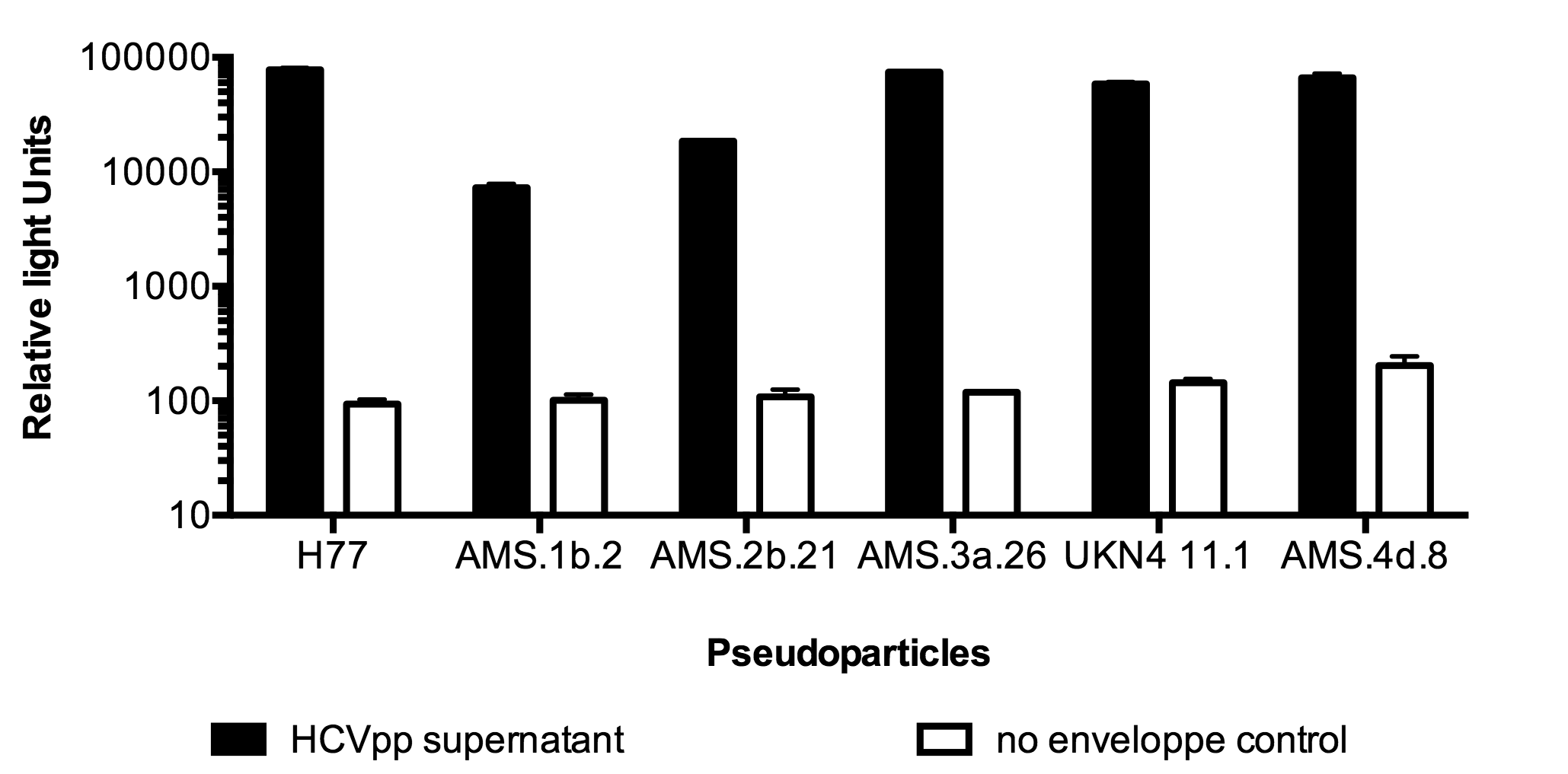

Supplement: S1 Fig — HCVpp carrying HCV envelope proteins from genotypes 1 to 4 were kept for 1 hour at 37°C before being added to Huh-7 cells. To determine the background infectivity of the assay, supernatant of 293T cells transfected only with phCMV-gag/pol and phCMV-Luciferase (no envelope glycoprotein control) was used. The mean value of a triplicate experiment is shown and the errors bars represent one standard deviation (SD). (TIFF) [file pone.0165047.s001.tiff]

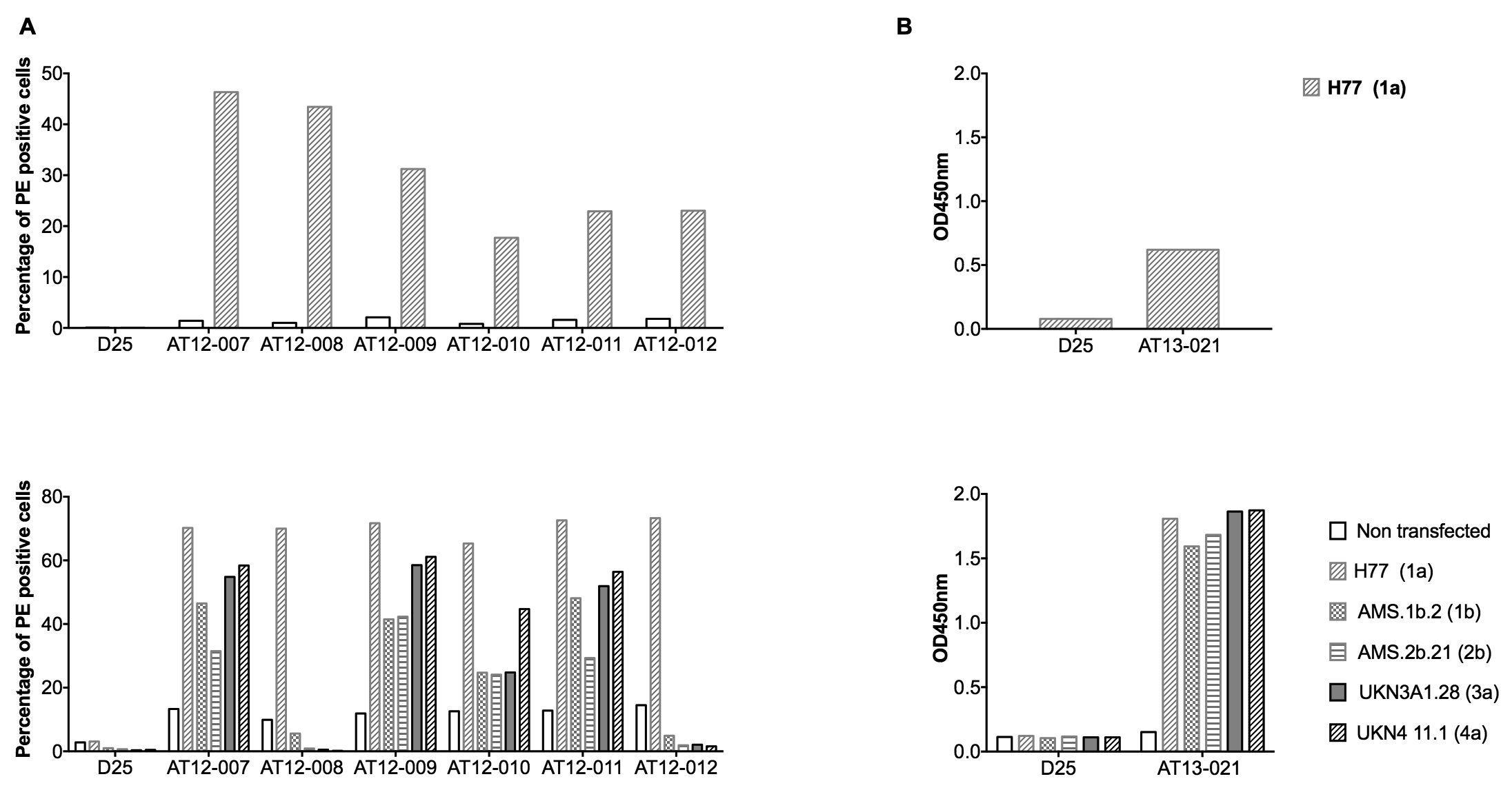

Supplement: S2 Fig — B cells specific for E1E2 were isolated using two strategies. (A) B cell supernatants were screened for binding to 293T cells expressing E1E2 by flow cytometry. E1E2 transfected 293T cells were permeabilized before adding B cell supernatants containing antibodies. First, B cell supernatant were screened for binding to 293T cells expressing E1E2 from gt 1a (H77) (top panel). Then, positive B cell supernatants were tested for binding E1E2 from genotype 1 to 4 (bottom panel). Non-transfected expressing 293T cells were used as control for non-E1E2 specific binding. (B) B cells specific for E1E2 were isolated with two rounds of cell sorting with E2 (AMS.2b.21, genotype 2b) followed by E2 from H77, genotype 1a. First, B cell supernatant of AT13-021 was tested binding on gt1a H77 E2 ELISA (top panel). Supernatant containing AT13-021 was tested for binding to E1E2 proteins derived from genotype 1 to 4 by ELISA (bottom panel). D25 was used as isotype control. (TIF) [file pone.0165047.s002.tif]

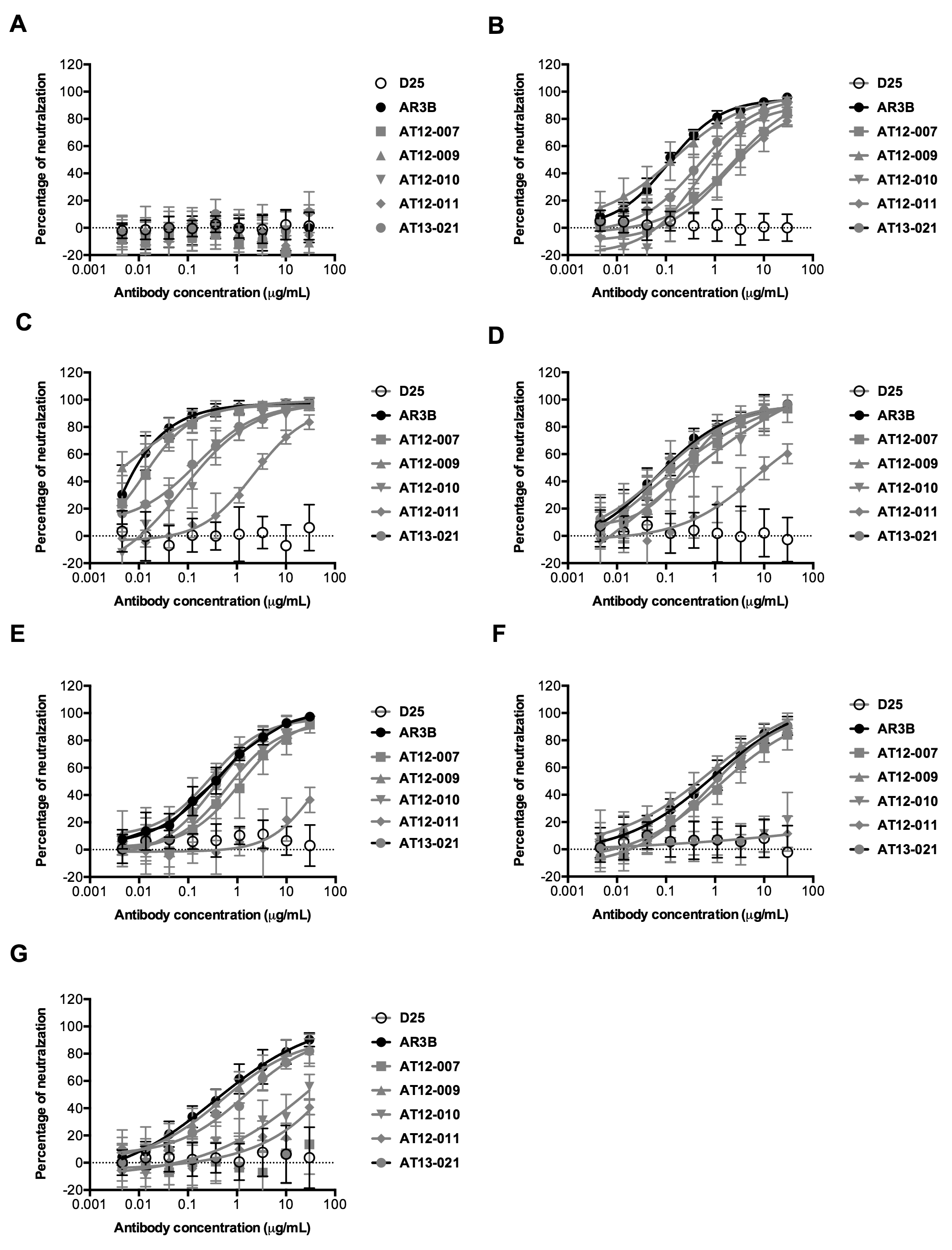

Supplement: S3 Fig — HCV antibody neutralizing activity was determined by pre-incubation of (A) VSV-G pp or HCVpp from isolates (B) H77 (genotype 1a), (C) AMS.1b.2 (genotype 1b), (D) AMS.2b.21 (genotype 2b), (E) AMS.3a.26 (genotype 3a), (F) UKN4.11.1 (genotype 4) and (G) AMS.4d.8 (gt4d) with antibodies (50 μg/mL to 0.0008 μg/mL) before being added to Huh-7 cells. The mean value of two triplicate experiments is shown and the errors bars represent one standard deviation (SD). (TIFF) [file pone.0165047.s003.tiff]

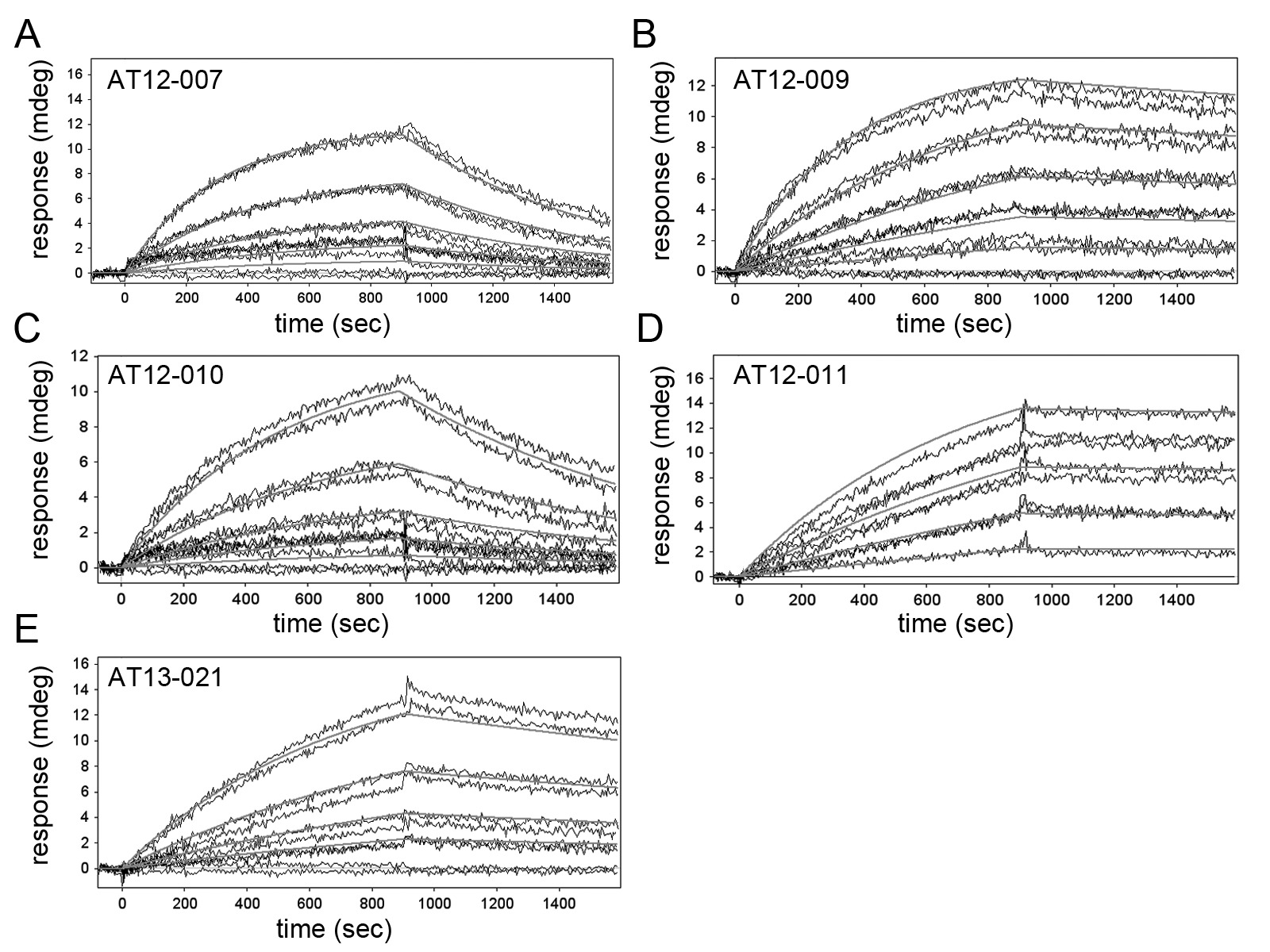

Supplement: S4 Fig — A concentration series of H77 derived E2-his (0.1–2.0 μg/mL) is injected in duplicate over an antibody-coated SPR chip. SPR data is shown as black curve. SPR curves are fit to a 1:1 binding model to obtain kinetic constants; curve fits are shown as grey lines. (TIF) [file pone.0165047.s004.tif]

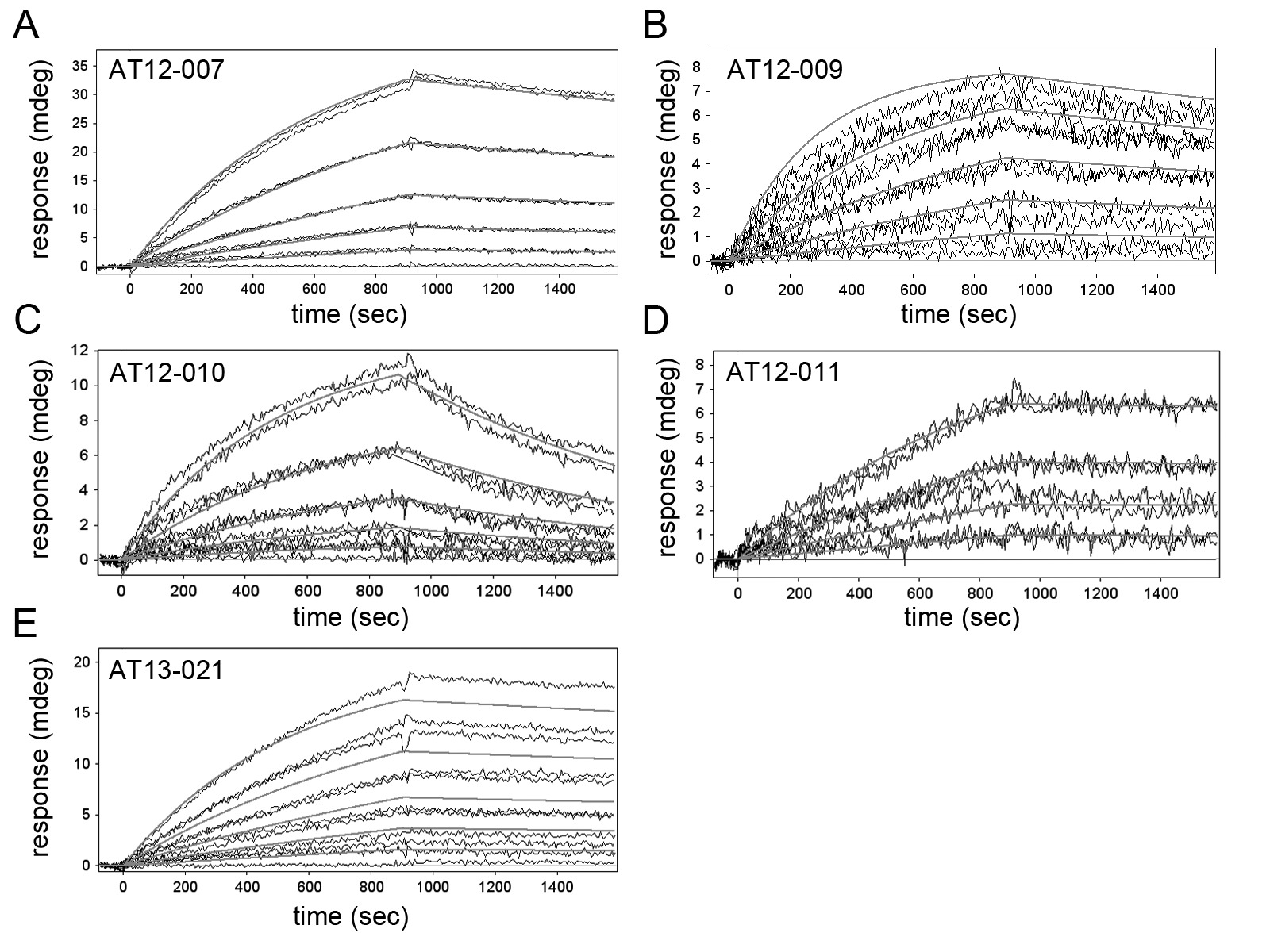

Supplement: S5 Fig — A concentration series of E2-his from genotype 2b isolate AMS.2b.21 (0.1–2.0 μg/mL) is injected in duplicate over an antibody-coated SPR chip. SPR data is shown as black curve. SPR curves are fit to a 1:1 binding model to obtain kinetic constants; curve fits are shown as grey lines. (TIF) [file pone.0165047.s005.tif]

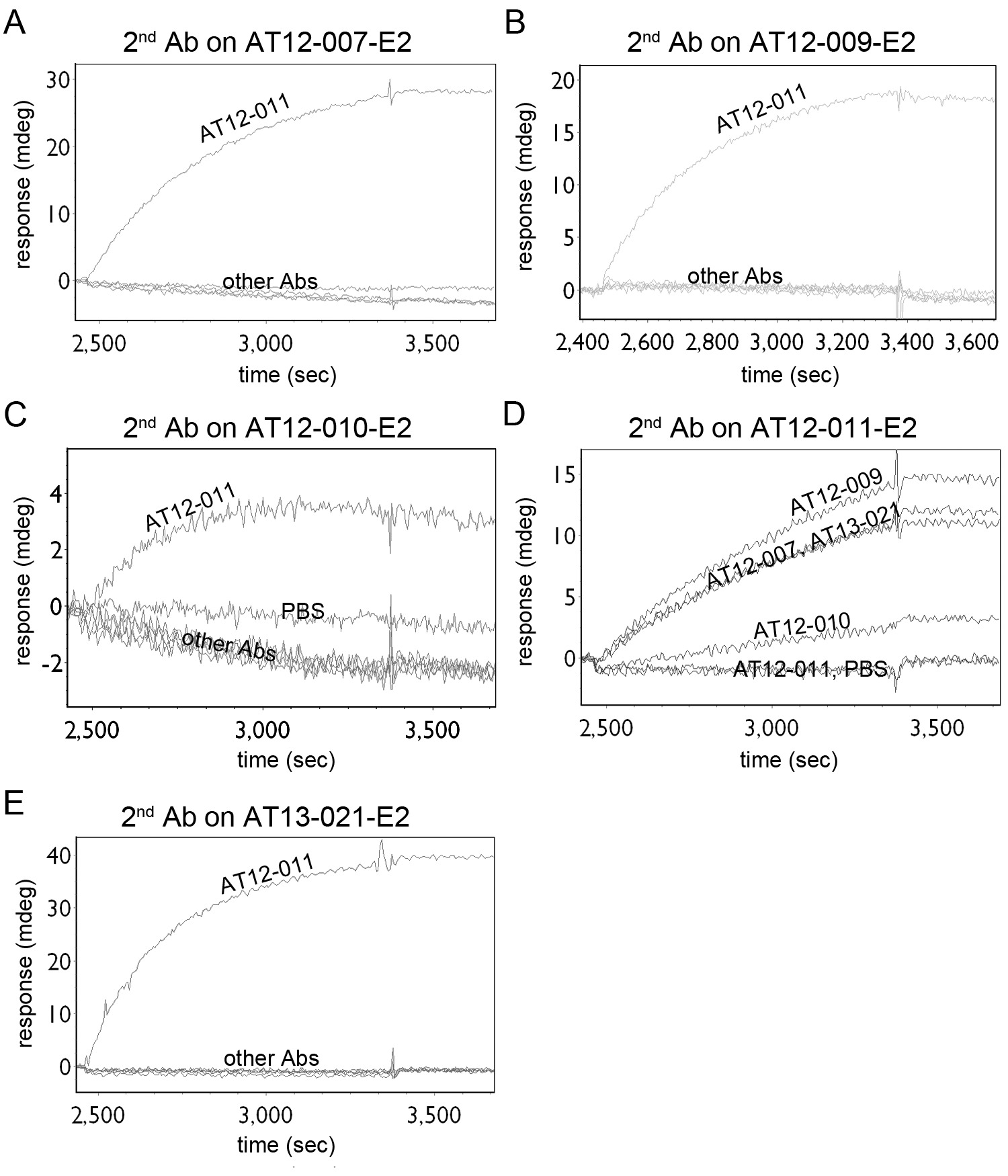

Supplement: S6 Fig — E2-his (2.0 μg/mL) is first injected over an antibody-coated SPR chip. After binding E2-his, a second antibody is injected. If the second antibody binds to the antibody-E2 complex, this indicates that the second antibody has a different epitope than the first antibody. (TIF) [file pone.0165047.s006.tif]
